# Correlates of HIV-1 Genital Shedding in Tanzanian Women

**DOI:** 10.1371/journal.pone.0017480

**Published:** 2011-03-01

**Authors:** Clare Tanton, Helen A. Weiss, Jerome Le Goff, John Changalucha, Mary Rusizoka, Kathy Baisley, Dean Everett, David A. Ross, Laurent Belec, Richard J. Hayes, Deborah Watson-Jones

**Affiliations:** 1 Department of Infectious Disease Epidemiology, London School of Hygiene and Tropical Medicine, London, United Kingdom; 2 National Institute for Medical Research (NIMR) Mwanza Centre, NIMR, Mwanza, Tanzania; 3 Laboratoire de Microbiologie, Hôpital Saint Louis, Paris, France; 4 African Medical and Research Foundation (AMREF) Lake Zone Office, AMREF, Mwanza, Tanzania; 5 Department of Clinical Research, London School of Hygiene and Tropical Medicine, London, United Kingdom; 6 Laboratoire de virologie, Hôpital Européen Georges Pompidou, et Université Paris Descartes, Paris, France; University of Cape Town, South Africa

## Abstract

**Background:**

Understanding the correlates of HIV shedding is important to inform strategies to reduce HIV infectiousness. We examined correlates of genital HIV-1 RNA in women who were seropositive for both herpes simplex virus (HSV)-2 and HIV-1 and who were enrolled in a randomised controlled trial of HSV suppressive therapy (aciclovir 400 mg b.i.d vs. placebo) in Tanzania.

**Methodology:**

Samples, including a cervico-vaginal lavage, were collected and tested for genital HIV-1 and HSV and reproductive tract infections (RTIs) at randomisation and 6, 12 and 24 months follow-up. Data from all women at randomisation and women in the placebo arm during follow-up were analysed using generalised estimating equations to determine the correlates of cervico-vaginal HIV-1 RNA detection and load.

**Principal Findings:**

Cervico-vaginal HIV-1 RNA was detected at 52.0% of 971 visits among 482 women, and was independently associated with plasma viral load, presence of genital ulcers, pregnancy, bloody cervical or vaginal discharge, abnormal vaginal discharge, cervical ectopy, *Neisseria gonorrhoeae*, *Chlamydia trachomatis*, *Trichomonas vaginalis*, an intermediate bacterial vaginosis score and HSV DNA detection. Similar factors were associated with genital HIV-1 RNA load.

**Conclusions:**

RTIs were associated with increased presence and quantity of genital HIV-1 RNA in this population. These results highlight the importance of integrating effective RTI treatment into HIV care services.

## Introduction

Plasma HIV-1 RNA viral load (PVL) is a major determinant of HIV transmission [1] and studies report a strong correlation between PVL and both genital HIV-1 detection and viral load. However, genital HIV shedding is more variable than PVL and is also influenced by local factors [2]. A recent systematic review and meta-analysis summarised the association between genital tract infections and genital HIV-1 [3] and reported that, in addition to PVL, genital HIV-1 detection is associated with both symptomatic and asymptomatic reproductive tract infections (RTIs). Treatment studies support these findings with a reduction in genital HIV-1 RNA load following treatment for *Neisseria gonorrhoeae*, *Chlamydia trachomatis*, *Trichomonas vaginalis* and *Candida albicans* [4], [5]. Suppressive therapy for herpes simplex virus type 2 (HSV-2) has been shown to reduce both detection and quantity of genital HIV-1 RNA over periods of upto three months in several randomised controlled trials (RCTs) [6], [7], [8], [9], [10], [11]. Episodic therapy for HSV-2 has shown less consistent results [12], [13], [14].

In addition to RTIs, hormonal contraceptives and pregnancy have been associated with genital HIV-1 DNA detection [15], [16], [17] and more recently, a dose-response association between vaginal cleansing and HIV-1 RNA shedding has been reported [18].

In this paper we report factors associated with presence and quantity of cervico-vaginal HIV-1 RNA in HIV-infected women working in bars, guesthouses and other recreational facilities, who were enrolled in an individually randomised, placebo-controlled trial of herpes suppressive therapy in Tanzania.

## Materials and Methods

Following screening for HSV-2 antibodies, eligible HSV-2 seropositive women working in food or recreational facilities in 19 communities in northwestern Tanzania were invited to participate in a randomised double-blind placebo-controlled trial of acyclovir (400 mg b.i.d) (ISRCTN35385041). Study procedures, including those for enrolment and eligibility criteria, have been described in detail previously [19], [20]. Both HIV-1 seronegative and seropositive women were enrolled to examine the effect of acyclovir on HIV acquisition and cervico-vaginal HIV-1 shedding, respectively. There was no evidence of an impact of acyclovir 400 mg b.i.d on either HIV acquisition or genital HIV-1 RNA shedding [20], [21].

For the purposes of the study reported here, the study population was restricted to the women who were HIV-1 seropositive at enrolment to the trial.

### Ethics statement

The trial protocol was approved by the London School of Hygiene & Tropical Medicine Ethics Committee and the Medical Research Coordinating Committee of Tanzania. Women provided written or fingerprinted consent to participate [20], [21].

### Study participants and trial procedures

At enrolment, venous blood samples were taken and participants were asked about their sexual behaviour, vaginal cleansing practices, RTI symptoms and history of symptoms consistent with HSV-2 infection. Vaginal cleansing was defined as inserting fingers (dry or wet) or any other substances (water, other liquids or items like cloths) into the vagina specifically to clean the vagina. During a genital examination, swabs were taken from the vagina and cervix for the diagnosis of RTIs. An additional swab was taken from any genital ulcers. Prior to the cervical swab, genital secretions were collected by cervico-vaginal lavage (CVL) using 3 ml of phosphate-buffered saline [22]. Follow-up visits were conducted at the study clinic every three months for 12 or 30 months depending on the woman's date of enrolment. A clinical examination was carried out every 6 months, and genital swabs and a CVL were taken at 6, 12 and 24 month follow-up visits. Women were asked to avoid vaginal cleansing within the 24 hours before attending the clinic.

At each visit, RTIs were treated syndromically. Women were offered treatment at their next scheduled follow-up visit for any asymptomatic sexually transmitted infections (STIs) identified through laboratory testing of the cervical and vaginal swabs. At every clinic visit, all women were offered family planning, condoms, risk reduction counselling and voluntary counselling and testing for HIV. Antiretroviral therapy (ART) became available in selected study communities during the trial and HIV-positive women who had asked to know their status were referred to these clinics for assessment and were given help with transport where requested. Funding was provided to allow participants to travel to their nearest care and treatment centre for 12 to 18 months after the trial.

### Laboratory analyses

Sera collected during screening and randomisation were tested for HSV-2 and HIV-1 antibodies by ELISA as described previously [20]. Randomisation sera were tested for syphilis using a quantitative rapid plasma reagin (RPR) test (Immutrep RPR, Omega Diagnostics Ltd, Alva, UK) and the *Treponema pallidum* particle agglutination assay (Serodia TPPA, Fujirebio Inc, Tokyo, Japan). RPR positive, TPPA negative/indeterminate samples were tested by a fluorescent treponemal antibody assay (FTA, Trepo-Spot IF, bioMérieux, Marcy l'Etoile, France). Follow-up sera were tested by RPR. New RPR positive samples were tested by TPPA and FTA if appropriate.

Vaginal and cervical swabs were tested for other RTIs [19], [20], [23]. Heat-fixed, gram-stained vaginal smears were examined by light microscopy for evidence of bacterial vaginosis using the Ison-Hay grading scheme [24]. *Trichonomas vaginalis* was diagnosed through examination using light miscroscopy of vaginal wet preparations and cultures (InPouch TV, BioMed Diagnostics, San Jose, California, USA). Vaginal wet preparations were also examined for evidence of *Candida albicans* hyphae and spores. Cervical swabs were tested by polymerase chain reaction (PCR) for *Neisseria gonorrhoeae* and *Chlamydia trachomatis* (Amplicor CT/NG PCR assay, Roche Diagnostics, Branchburg, NJ, USA). Genital ulcer swabs were tested for *T. pallidum*, HSV and *Haemophilus ducreyi* by PCR.

NucliSens® miniMAG and subsequently easyMAG (bioMérieux) was used to extract nucleic acids from plasma and CVL supernatants. Extractions of CVL cell pellets used the Qiagen QIAamp DNA Blood Mini Kit (Qiagen, Courtabeouf, France).

HIV-1 RNA was quantified in CVL supernatants and plasma by in-house Real-Time PCR as described previously [25] but using a shorter forward primer. HSV DNA was quantified in CVL supernatants by in-house real-time PCR [26]. Thresholds for quantitation were 360 copies/ml for cervico-vaginal HIV-1 RNA, 300 copies/ml for cervico-vaginal HSV DNA and 300 copies/ml for plasma HIV-1 RNA. Nucleic acids extracted from the CVL pellet were used to detect Y-chromosome DNA (Quantifiler™ Y Human Male DNA Quantification kit, Applied Biosystems, Courtaboeuf, France) as evidence for contamination with male secretions. Quantifications were carried out using the Applied Biosystems 7300 Real-time PCR System (Applied Biosystems).

### Statistical methods

Data were double-entered and verified in Dbase IV (dataBased Intelligence) and statistical analyses were carried out using Stata 10.0 (Stata Corporation, College Station, Texas, USA).

Factors associated with cervico-vaginal HIV-1 RNA were examined using data from all women at randomisation and women in the placebo arm at 6, 12 and 24 month follow-up visits. Women in the aciclovir arm were excluded since other studies have shown that suppressive therapy with aciclovir can reduce plasma and genital HIV RNA levels. Data were censored when women started ART. Separate analyses were carried out of factors associated with (i) detection of HIV-1 RNA and (ii) HIV-1 RNA load (log_10_ transformed) among those with detectable HIV-1 RNA. Factors associated with HIV-1 RNA load were only examined in those with detectable HIV-1 RNA since there were a large number of women with no detectable shedding and replacing undetectable values with zero or half the limit of quantification would have resulted in a highly skewed distribution. Logistic and linear regression models with general estimating equations (GEE) and an exchangeable correlation matrix were used to assess factors associated with detection and quantity of cervico-vaginal HIV-1 RNA, respectively. PVL was hypothesised *a priori* to be strongly associated with cervico-vaginal HIV-1 RNA and was adjusted for in all analyses in order to examine local correlates of HIV shedding. Statistical significance was assessed using the Wald test. For both the analysis of correlates of HIV-1 RNA detection and the analysis of correlates of HIV-1 RNA load, factors associated at p<0.15 were included in a multivariable model and retained in the final model if they were independently associated with the outcome (p<0.1). RTIs were defined on the basis of laboratory results, not syndromic diagnoses.

Further linear regression analyses of the association between HIV-1 RNA and HSV DNA viral loads were conducted for: i) all women with detectable HSV DNA by assigning a value of half the threshold of quantification for women with undetectable HIV-1 RNA (to examine the overall association between HSV and HIV shedding, irrespective of whether HIV shedding was detected); and ii) in women with both detectable HSV DNA and detectable HIV-1 RNA. Results of analyses restricted to samples without visible blood or detectable Y-chromosome, to avoid misclassification of results by contamination with virus originating from the blood or a male partner, are also shown.

## Results

### Characteristics of the study population

A flowchart of enrolment and full details of the study population have been published previously [21]. In total, 484 dually HSV-2/HIV-1 seropositive women were enrolled and randomised to aciclovir (253 women) or placebo (231 women). Data were censored when women started ART and one woman was excluded from analysis as she was on ART at enrolment. Data were analysed from a total of 971 visits (482 of the eligible 483 women at enrolment and 489 of 631 (77%) eligible follow-up visits i.e. a total of 87% of eligible visits). These 489 follow-up visits were made by 204 women on placebo. Most missing data on booked visits (134/143 visits; 94%) were due to women not attending the visit. The remainder (9/143; 6%) resulted from missing outcome or PVL data.

At enrolment, the median age of participants was 28 years, 18.6% were married or living as married and 46.1% worked as local food handlers. The median reported age at first sex was 16 years and 38.6% of participants reported 10 or more lifetime sexual partners. Sex in the last year during menstruation was reported by 15.9% of women. Most women (61.4%) reported practising vaginal cleansing more than once a day. A history of genital ulcers was reported by 31.1% of women and 24.1% reported an episode of genital ulceration within the last year.

Laboratory-confirmed RTIs were prevalent at baseline, especially bacterial vaginosis (BV) and *T. vaginalis* (70.1% and 35.5% of women respectively), but also *N. gonorrhoeae* (7.1%), *C. trachomatis* (6.0%) and *C. albicans* (10.6%). Of the 204 women attending at least one follow-up visit, 83.3% had BV during at least one follow-up visit, 38.2% had *T. vaginalis*, 11.8% had *N. gonorrhoeae*, 11.3% had *C. trachomatis* and 17.2% had *C. albicans*.

### Factors associated with cervico-vaginal HIV-1 RNA detection

Cervico-vaginal HIV-1 RNA was detected at 505 visits (52.0%). Tables 1 & 2 show the association between HIV-1 RNA detection and behavioural and clinical factors. HIV-1 RNA detection was not increased in those using hormonal contraceptives, and there was no association with vaginal cleansing. As expected, prevalence of cervico-vaginal HIV-1 RNA detection was strongly associated with PVL increasing from 6.5% among those with undetectable PVL to 87.4% among those with PVL of 5.5 log_10_ copies/ml or greater. After adjusting for PVL, cervico-vaginal HIV-1 RNA detection was not associated with a history of genital ulceration but was associated with being pregnant, presence of genital ulcers on examination, abnormal or bloody cervical or vaginal discharge and cervical ectopy (Tables 1 & 2). Biological factors associated with cervico-vaginal HIV-1 RNA detection were *N. gonorrhoeae*, *C. trachomatis*, *T. vaginalis*, an intermediate BV score and HSV DNA detection (Table 3).

**Table 1:** Behavioural characteristics and association with cervico-vaginal HIV-1 RNA detection (in 482 women at 971 visits) and HIV-1 RNA load.

	No. of visits	HIV-1 RNA detected No. (%)	PVL-adjusted OR* (95%CI)	Mean HIV RNA load (log_10_ copies/ml)	PVL-adjusted regression coefficient* (95%CI)
**Currently using hormonal contraceptive**					
			P = 0.08		P = 0.41
No	514	268 (52.1)	1	3.64	-
Yes	436	224 (51.4)	1.03 (0.76, 1.38)	3.65	0.05 (−0.07, 0.18)
Pregnant	21	13 (61.9)	2.91 (1.15, 7.37)	3.48	−0.13 (−0.47, 0.19)
**Frequency of vaginal cleansing (per/day)** †					
			P = 0.96		P = 0.02
0	324	172 (53.1)	1	3.69	-
1	51	26 (51.0)	1.00 (0.57, 1.76)	3.45	−0.33 (−0.63, −0.03)
2	229	122 (53.3)	0.99 (0.63, 1.55)	3.80	0.04 (−0.13, 0.21)
3	291	151 (51.9)	0.90 (0.61, 1.32)	3.53	−0.19 (−0.35, −0.02)
≥4	74	32 (43.2)	0.84 (0.45, 1.55)	3.50	−0.16 (−0.41, 0.10)
**Time since last vaginal cleansing**					
			P = 0.49		P = 0.41
>12 hours	503	262 (52.1)	1	3.60	-
7–12 hours	138	74 (53.6)	1.13 (0.74, 1.73)	3.66	0.05 (−0.12, 0.22)
4–6 hours	151	74 (49.0)	0.94 (0.65, 1.37)	3.76	0.15 (−0.04, 0.34)
1–3 hours	179	95 (53.1)	1.29 (0.87, 1.92)	3.64	0.08 (−0.09, 0.24)
**Cleansed with**					
			P = 0.46		P = 0.30
No cleansing	503	262 (52.1)	1	3.60	-
Water	187	96 (51.3)	0.98 (0.67, 1.43)	3.74	0.09 (−0.05, 0.26)
Soap & water	281	147 (52.3)	1.22 (0.88, 1.70)	3.64	0.09 (−0.05, 0.23)

P-values for regression coefficients calculated using Wald test.

*Adjusted for PVL as a categorical variable;

† Recorded at enrolment.

**Table 2:** Behavioural characteristics and association with cervico-vaginal HIV-1 RNA detection (in 482 women at 971 visits) and HIV-1 RNA load.

	No. of visits	HIV-1 RNA detected No. (%)	PVL-adjusted OR* (95%CI)	Mean HIV RNA load (log_10_ copies/ml)	PVL-adjusted regression coefficient* (95%CI)
**Self reported frequency of ulcers** †					
			P = 0.30		P = 0.83
No history of ulcers	677	330 (48.7)	1	3.62	-
Only once/twice ever	77	40 (52.0)	0.83 (0.50, 1.38)	3.75	0.07 (−0.15, 0.30)
Once/twice per year	67	39 (58.2)	1.22 (0.60, 2.46)	3.78	0.07 (−0.20, 0.34)
Every 3 months	67	47 (70.2)	1.69 (0.97, 2.93)	3.56	−0.09 (−0.31, 0.13)
Monthly	76	45 (59.2)	1.26 (0.64, 2.49)	3.67	−0.01 (−0.26, 0.23)
**Self reported episode of GUD in last year** †					
			P = 0.35		P = 0.66
No	733	362 (49.4)	1	3.64	-
Yes	233	140 (60.1)	1.19 (0.82, 1.72)	3.65	−0.03 (−0.17, 0.11)
**Genital ulcers on examination**					
			P = 0.05		P = 0.57
No	940	482 (51.3)	1	3.64	-
Yes	31‡	23 (74.2)	2.23 (0.99, 5.02)	3.72	−0.06 (−0.26, 0.14)
**Blisters on examination**					
			P = 0.56		P = 0.49
No	958	495 (51.7)	1	3.64	-
Yes	13	10 (76.9)	1.43 (0.44, 4.63)	3.74	−0.17 (−0.65, 0.31)
**Cervical discharge**					
			P = 0.0002		P = 0.002
None/Normal	656	317 (48.3)	1	3.59	-
Present§	176	101 (57.4)	1.33 (0.93, 1.91)	3.65	0.03 (−0.13, 0.18)
Bloody	139	87 (62.6)	2.48 (1.59, 3.84)	3.81	0.25 (0.11, 0.38)
**Vaginal discharge**					
			P<0.0001		P = 0.002
Normal	477	205 (43.0)	1	3.56	-
Abnormal∥	355	208 (58.6)	1.90 (1.39, 2.59)	3.65	0.12 (−0.02, 0.25)
Bloody	139	92 (66.2)	3.74 (2.38, 5.88)	3.81	0.29 (0.15, 0.43)
**PID diagnosis**					
			P = 0.27		P = 0.32
No	866	440 (50.8)	1	3.62	-
Yes	105	65 (61.9)	1.30 (0.81, 2.09)	3.76	0.10 (−0.09, 0.29)
**Cervical ectopy**					
			P = 0.02		P = 0.13
No	865	437 (50.5)	1	3.61	-
Yes	106	68 (64.2)	1.72 (1.07, 2.75)	3.81	0.16 (−0.05, 0.37)

P-values for regression coefficients calculated using Wald test.

*Adjusted for PVL as a categorical variable;

† Recorded at enrolment;

‡ Of 30 ulcer swabs taken 19 (63%) had no aetiology, 10 (33%) were HSV-2 and 1 (3%) was syphilis;

§ White, cream or purulent or malodorous;

∥ Curdlike, white or purulent.

**Table 3:** Biological characteristics and association with cervico-vaginal HIV-1 RNA detection (in 482 women at 971 visits) and HIV-1 RNA load.

	No. of visits	HIV-1 RNA detected No. (%)	PVL-adjusted OR* (95%CI)	Mean HIV RNA load (log_10_ copies/ml)	PVL-adjusted regression coefficient* (95%CI)
***N gonorrhoeae***					
			P = 0.02		P = 0.05
No	911	467 (51.3)	1	3.62	-
Yes	59	38 (64.4)	2.02 (1.13, 3.59)	3.84	0.25 (−0.00, 0.51)
***C trachomatis***					
			P = 0.05		P = 0.73
No	915	469 (51.3)	1	3.64	-
Yes	54	36 (66.7)	1.99 (0.99, 3.99)	3.66	0.04 (−0.17, 0.24)
***T. vaginalis***					
			P = 0.008		P = 0.01
No	696	340 (48.9)	1	3.60	-
Yes	273	164 (60.1)	1.56 (1.12, 2.17)	3.72	0.16 (0.03, 0.29)
***C. albicans***					
			P = 0.79		P = 0.57
No	878	453 (51.6)	1	3.64	−
Yes	91	51 (56.0)	1.07 (0.65, 1.77)	3.63	−0.05 (−0.24, 0.13)
**Bacterial vaginosis**					
			P = 0.01		P = 0.003
Normal	113	59 (52.2)	1	3.45	-
Intermediate	192	121 (63.0)	1.69 (1.01, 2.83)	3.73	0.31 (0.15, 0.48)
BV	626	306 (48.9)	0.89 (0.58, 1.38)	3.64	0.21 (0.06, 0.35)
Slides unclassifiable	40	19 (47.5)	0.91 (0.44, 1.88)	3.68	0.20 (−0.09, 0.50)
**High-titre active syphilis** †					
			P = 0.21		P = 0.56
No/past infection	927	480 (51.8)	1	3.65	-
Yes	44	25 (56.8)	1.53 (0.79, 2.98)	3.55	−0.08 (−0.37, 0.20)
**Y-PCR positive**					
			P = 0.12		P = 0.02
No	753	407 (54.1)	1	3.68	-
Yes	209	95 (45.5)	0.76 (0.54, 1.08)	3.48	−0.17 (−0.32, −0.03)
**HSV DNA detected**					
			P = 0.04		P = 0.84
No	842	419 (49.8)	1	3.62	-
Yes	129	86 (66.7)	1.58 (1.03, 2.41)	3.72	−0.02 (−0.18, 0.15)
**PVL (log copies/ml)**					
			P<0.0001		P<0.0001
Undetectable	92	6 (6.5)	0.22 (0.10, 0.51)	3.83	0.55 (−0.17, 1.26)
2.45–4.0	215	62 (28.8)	1	3.18	-
4.0–<5.0	345	183 (53.0)	2.65 (1.79, 3.92)	3.46	0.26 (0.11, 0.41)
5.0–<5.5	192	143 (74.5)	6.19 (3.98, 9.64)	3.77	0.56 (0.39, 0.74)
≥5.5	127	111 (87.4)	15.30 (8.38, 27.92)	4.02	0.83 (0.63, 1.03)

P-values for regression coefficients calculated using Wald test.

*Adjusted for PVL as a categorical variable;

† new RPR positive with titre ≥8 & TPPA/FTA positive or RPR positive with titre ≥8 and previously RPR positive.

In the multivariable model (Table 4), HIV-1 RNA detection was independently associated with pregnancy, the presence of genital ulcers, bloody cervical discharge, cervical ectopy, RTIs (*N. gonorrhoeae*, *C. trachomatis*, *T. vaginalis*, an intermediate BV score) and HSV DNA detection (adjusted odds ratio (aOR) = 1.55, 95%CI 0.99–2.42). A PVL of 5.5 log copies/ml was associated with 15.57 times increased odds of HIV-1 RNA detection compared with a PVL of 2.45–4.0 log copies/ml. Vaginal discharge was excluded due to collinearity with cervical discharge but associations were similar in a model including vaginal discharge instead of cervical discharge and both abnormal (aOR = 1.74, 95%CI 1.26–2.41) and bloody (aOR = 3.80, 95%CI 2.37–6.10) vaginal discharge were associated with increased odds of HIV-1 RNA detection (results not shown).

**Table 4:** Factors independently associated with cervico-vaginal HIV-1 RNA detection (in 482 women at 967 visits).

	No. of visits	HIV-1 RNA detected No. (%)	Adjusted OR (95%CI)*
**Currently using hormonal contraceptive**			
			P = 0.07
No	512	268 (52.3)	1
Yes	434	223 (51.4)	1.07 (0.79, 1.46)
Pregnant	21	13 (61.9)	3.17 (1.19, 8.46)
**Genital ulcers on examination**			
			P = 0.10
No	936	481 (51.4)	1
Yes	31	23 (74.2)	2.01 (0.88, 4.60)
**Cervical discharge**			
			P = 0.0002
None/Normal	653	316 (48.4)	1
Present †	175	101 (57.7)	1.22 (0.83, 1.80)
Bloody	139	87 (62.6)	2.59 (1.63, 4.12)
**Cervical ectopy**			
			P = 0.04
No	861	436 (50.6)	1
Yes	106	68 (64.2)	1.68 (1.02, 2.76)
***N gonorrhoeae***			
			P = 0.07
No	908	466 (51.3)	1
Yes	59	38 (64.4)	1.80 (0.96, 3.39)
***C trachomatis***			
			P = 0.08
No	913	468 (51.3)	1
Yes	54	36 (66.7)	1.90 (0.92, 3.95)
***T. vaginalis***			
			P = 0.01
No	695	340 (48.9)	1
Yes	272	164 (60.3)	1.58 (1.11, 2.25)
**Bacterial vaginosis**			
			P = 0.03
Normal	113	59 (52.2)	1
Intermediate	191	121 (63.4)	1.39 (0.83, 2.34)
BV	623	305 (49.0)	0.75 (0.47, 1.19)
Unclassifiable	40	19 (47.5)	0.75 (0.45, 1.58)
**HSV DNA detected**			
			P = 0.06
No	839	419 (49.9)	1
Yes	128	85 (66.4)	1.55 (0.99, 2.42)
**PVL (log copies/ml)**			
			P<0.0001
Undetectable	92	6 (6.5)	0.20 (0.08, 0.50)
2.45–4.0	213	62 (29.1)	1
4.0–<5.0	344	183 (53.2)	2.98 (1.98, 4.48)
5.0–<5.5	191	142 (74.4)	6.83 (4.27, 10.93)
≥5.5	1270	111 (87.4)	15.57 (8.47, 28.61)

*Adjusted for hormonal contraceptive use, ulcers, cervical discharge, cervical ectopy, *N. gonorrhoeae*, *C. trachomatis*, *T. vaginalis*, BV, HSV shedding and PVL;

† White, cream or purulent.

### Correlates of cervico-vaginal HIV-1 RNA load

The correlates of cervico-vaginal HIV-1 RNA load were examined in the 505 visits with detectable HIV-1 RNA. The quantity of cervico-vaginal HIV-1 RNA detected ranged from 2.55 to 6.58 log copies/ml. There was little association between behavioural variables and HIV-1 RNA load (Table 1). Among those with detectable HIV-1 RNA, genital viral load was associated (p<0.15) with vaginal cleansing, having either bloody cervical or vaginal discharge, cervical ectopy, *N. gonorrhoeae*, *T. vaginalis*, an intermediate BV grading and BV (Tables 2 & 3). There was no association with *C. trachomatis* or HSV DNA detection. Presence of Y-chromosome was associated with a reduced HIV-1 RNA load. With the exception of cervical ectopy, these factors were independently associated with HIV-1 RNA load in multivariate analysis (Table 5). Coefficients were similar when vaginal discharge was included instead of cervical discharge (results not shown).

**Table 5:** Factors independently associated with cervico-vaginal HIV-1 RNA load (in 311 women with detectable shedding at 499 visits).

	No. of visits	Mean HIV RNA load (95%CI) (log_10_ copies/ml)	Adjusted regression coefficient† (95%CI)
**Frequency of vaginal cleansing (per/day)**			
			P = 0.02
0	171	3.69 (3.57, 3.80)	-
1	26	3.45 (3.12, 3.79)	−0.34 (−0.63, −0.06)
2	121	3.79 (3.67, 3.91)	0.01 (−0.16, 0.19)
3	149	3.53 (3.41, 3.65)	−0.18 (−0.35, −0.01)
≥4	32	3.50 (3.26, 3.74)	−0.18 (−0.43, 0.06)
**Cervical discharge**			
			P = 0.003
None/Normal	311	3.59 (3.51, 3.67)	-
Present ‡	101	3.65 (3.50, 3.81)	−0.01 (−0.16, 0.13)
Bloody	87	3.81 (3.65, 3.97)	0.23 (0.09, 0.37)
***N gonorrhoeae***			
			P = 0.05
No	461	3.62 (3.56, 3.69)	-
Yes	38	3.84 (3.55, 4.13)	0.25 (−0.00, 0.51)
***T. vaginalis***			
			P = 0.007
No	336	3.60 (3.53, 3.68)	-
Yes	163	3.72 (3.60, 3.84)	0.17 (0.05, 0.30)
**Bacterial vaginosis**			
			P = 0.02
Normal	58	3.45 (3.30, 3.61)	-
Intermediate	120	3.73 (3.59, 3.86)	0.24 (0.08, 0.39)
BV	302	3.64 (3.55, 3.72)	0.12 (−0.02, 0.26)
Unclassifiable	19	3.68 (3.31, 4.05)	0.05 (−0.25, 0.36)
**Y-PCR positive**			
			P = 0.10
No	404	3.68 (3.61, 3.75)	-
Yes	95	3.48 (3.32, 3.63)	−0.12 (−0.26, 0.02)
**PVL (log copies/ml)**			
			P<0.0001
Undetectable	6	3.83 (2.69, 4.97)	0.66 (−0.00, 1.33)
2.45–4.0	62	3.18 (3.06, 3.31)	-
4.0–<5.0	180	3.45 (3.36, 3.54)	0.31 (0.16, 0.47)
5.0–<5.5	141	3.77 (3.65, 3.89)	0.61 (0.43, 0.79)
≥5.5	110	4.03 (3.88, 4.18)	0.88 (0.68, 1.07)

P values calculated using Wald test.

*Adjusted for all variables in the table.

† White, cream or purulent.

### Relationship between HIV-1 RNA and HSV DNA

**Figure 1:**
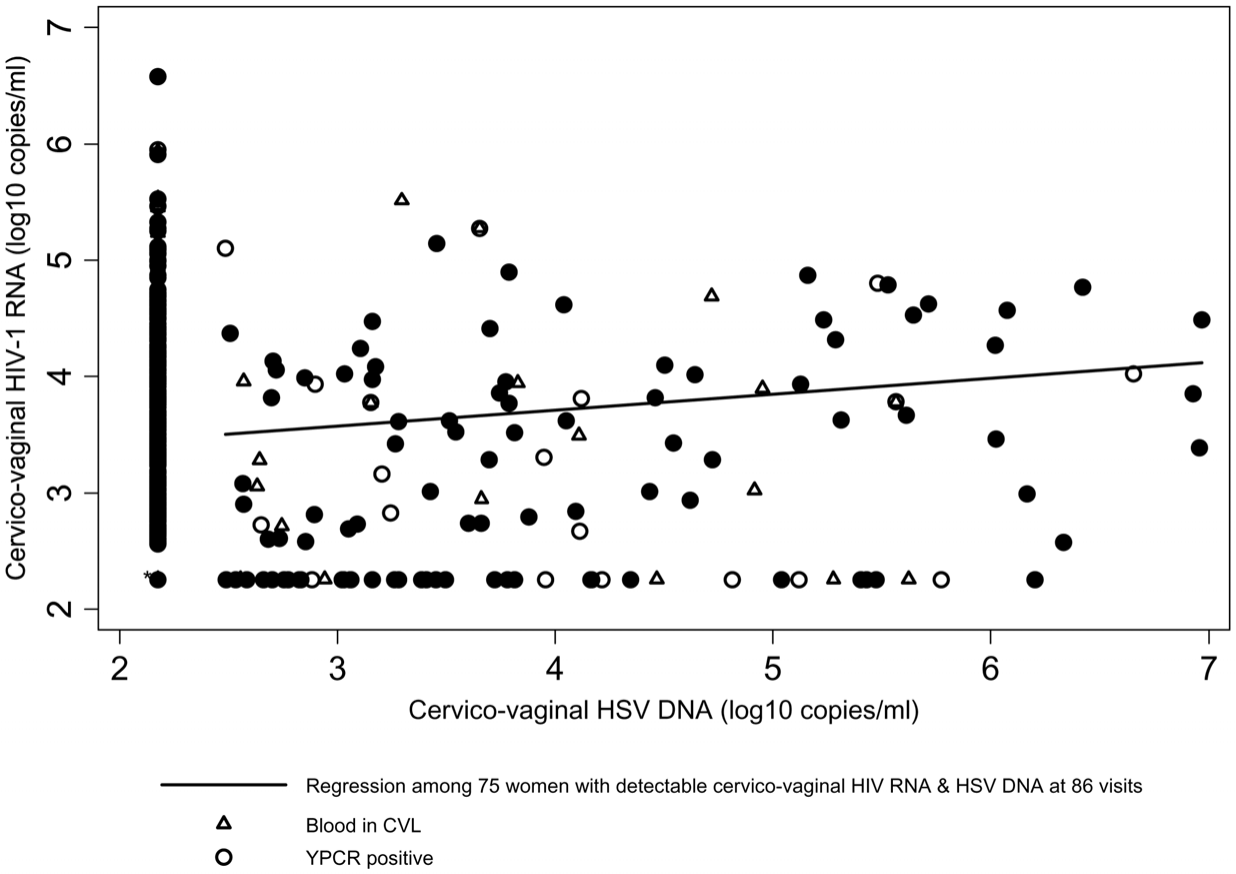
The relationship between cervico-vaginal HIV-1 RNA and HSV DNA among women with both viruses detected. Samples with visible blood or Y-PCR detected are indicated. Visits with undetectable HIV-1 RNA and HSV DNA were assigned a value of half the threshold of quantification. * This point marks 425 visits where HIV-1 RNA and HSV DNA were both undetectable.

Cervico-vaginal HSV DNA was detected at 129 visits (13.3%). The quantity of cervico-vaginal HSV DNA detected ranged from 2.49 to 6.97 log copies/ml. After adjusting for PVL, there was weak evidence for an association between HIV-1 RNA and HSV DNA viral loads at all visits when HSV was shed (Figure 1) when undetectable HIV-1 RNA was assigned a value of half the threshold of quantification (N = 129; adjusted regression coefficient  = 0.11, 95%CI −0.00, 0.22) and when restricted to samples without blood or Y-chromosome (N = 93; adjusted regression coefficient = 0.15, 95%CI 0.02, 0.27). Among the 86 samples with both cervico-vaginal HIV-1 RNA and HSV DNA detected, there was also weak evidence for an association (adjusted regression coefficient = 0.10, 95%CI −0.01, 0.22) and when restricted to samples without visible blood or Y-chromosome detected (N = 62; adjusted regression coefficient = 0.11, 95%CI −0.03, 0.24). Similar results were seen in both the analysis unadjusted for PVL and when women with genital ulcers were excluded.

## Discussion

In this study we examined correlates of genital HIV-1 RNA detection and viral load in a cohort of HSV-2 seropositive Tanzanian female facility workers enrolled in an HSV suppressive therapy trial. Genital HIV-1 RNA detection and viral load were associated with several clinical and biological factors in this cohort but there were no significant associations with behavioural variables measured in this study.

These findings are broadly consistent with other studies reporting an association between RTIs and genital HIV-1 RNA detection and viral load, especially with infections affecting the cervix [27], [28], [29], [30]. A meta-analysis reported significant associations between HIV-1 RNA detection and *N. gonorrhoeae*, *C. trachomatis*, vaginal discharge, cervical discharge and candidiasis and a non-significant association for both HSV DNA detection and genital ulcers [3]. Treatment studies support elevated viral loads with co-infection with *N. gonorrhoeae*, *C. trachomatis*, *T. vaginalis* and *C. albicans* [4], [5] and a reduction in viral load following treatment.

This study population was not selected based on genital symptoms or signs, but the high prevalence of RTIs and the association that was found between several RTIs and HIV shedding, although modest for some RTIs, highlights the potential for effective STI management to reduce HIV infectiousness by reducing HIV load in such populations. This is supported by observational data and one community randomised trial of STI treatment which reported 40% lower HIV incidence in communities that received improved syndromic management of STIs [31]. Other cluster trials of RTI treatment have produced apparently inconsistent results [32], [33], [34] and extensive work has been carried out to explore the reasons for these inconsistencies [35], [36], [37]. Although the association between HSV and HIV shedding in our study was weak, suppressive HSV therapy has been demonstrated to reduce HIV shedding [6], [7], [8], [9], [10], [11] even among women who had detectable shedding while on antiretrovirals [38], highlighting the importance of considering the impact of other local co-factors on HIV infectiousness. However, this impact of HSV suppressive therapy did not translate into an effect on HIV transmission in a recent study among HIV discordant couples despite a 0.25 log_10_ reduction in PVL [39]. The authors suggest that the reduction in genital HIV may have been insufficient to impact HIV transmission. More research is needed to understand the relationship between HIV shedding and transmission and, most importantly, what decrease in genital HIV would translate into a population level impact on HIV transmission. These are important questions for trials which will examine the impact of early initiation of antiretroviral therapy on population HIV acquisition.

Cervical and abnormal, vaginal discharge were associated with genital HIV in this analysis. Maintaining services that can provide good syndromic RTI management remains important, especially where laboratory diagnosis of RTIs is not readily available. Increased integration of RTI services into HIV care settings may be beneficial in widening service access to populations who may less readily engage with services.

In contrast to results of a recent meta-analysis [3], we observed a strong association between *T. vaginalis* and HIV-1 RNA detection. Although this may have arisen by chance, the moderate point estimate in our study suggests that the association may not be strong enough to have been detected in studies with a small sample size or low prevalence of *T. vaginalis*. Additionally, in our study, genital HIV-1 RNA detection was found to be associated with an intermediate BV score, but not with BV. Data from other studies are inconclusive [28], [40], [41] but *in vitro* studies have demonstrated that certain bacteria associated with BV induce HIV expression or activate the HIV long terminal repeat [42], [43] but further studies are needed to elucidate the association between BV and genital HIV shedding.

We found significantly increased odds of HIV-1 RNA detection with HSV DNA detection and genital ulceration. In the meta-analysis [3], both these estimates showed significant inter-study heterogeneity. Our findings and a study reporting a four-fold increase in HIV transmission probabilities with genital ulceration [44] highlight the potential importance of HSV treatment in HIV positive individuals, especially those who are immuno-compromised and who may have more frequent and more prolonged HSV recurrences [45]. Recent trials estimating the effect of episodic HSV therapy on genital HIV-1 shedding, however, have reported little effect [12], [13], [14]. Furthermore, the multi-country discordant couples study also found no reduction in HIV transmission with HSV suppressive therapy despite a 73% reduction in genital ulcers due to HSV-2 [39].

We did not find an association between HIV-1 RNA detection and hormonal contraceptive use, although this has been more frequently associated with HIV-infected cells [16], [17]. However, pregnancy was found to be associated with increased odds of HIV-1 RNA detection. Previous studies have found pregnancy to be associated with increased HIV-1 DNA detection [15], [46]. This association may be explained by hormonal or cervical changes but may also have been a chance finding.

Strengths of our study include the fact that we used CVLs to collect genital secretions so we were able to examine simultaneously the association between both cervical and vaginal infections and HIV shedding. The longitudinal design with repeated visits allowed a high powered analysis of correlates of HIV-1 shedding in this population. This is also a non-clinic population enabling examination of the importance of RTIs for HIV shedding in those not presenting to services. Unfortunately due to small sample sizes, we were unable to examine, for each RTI separately, whether HIV shedding was higher during symptomatic vs. asymptomatic infections, and therefore investigate a hypothesis proposed by Johnson et al. [3] that since leukocyte concentrations are more likely to be increased with symptomatic infections, these may have a greater effect on HIV shedding.

Our study confirms previous data on the importance of RTIs as a local correlate of HIV shedding, even in a non-clinic population. This highlights the importance of RTI treatment in HIV infected individuals. This may be especially important in populations with high prevalence of bacterial RTIs who are often those least able to access adequate treatment services. Increased integration of STI services into HIV care settings may also be beneficial. Further research is needed to understand the relationship between HIV shedding and HIV transmission.
